# Myocardial Infarction in Systemic Lupus Erythematosus: Incidence and Coronary Angiography Findings

**DOI:** 10.1177/0003319720985337

**Published:** 2021-01-08

**Authors:** Per Tornvall, Alexandra Göransson, Julia Ekman, Hans Järnbert-Pettersson

**Affiliations:** 1 Department of Clinical Science and Education Södersjukhuset, 27106 Karolinska Institutet, Stockholm, Sweden

**Keywords:** systemic lupus erythematosus, myocardial infarction, incidence, nonobstructive coronary arteries

## Abstract

An association between acute myocardial infarction (AMI) and systemic lupus erythematosus (SLE) has been suggested. The cause of AMI is presumed to be atherothrombosis. In the present study, the primary objective was to assess incident AMI cases and the secondary objective was to estimate the proportion of myocardial infarction with nonobstructive coronary arteries (MINOCA) in patients with SLE. All Swedish patients with SLE without AMI before 1996 (n = 4192) were followed for 20 years in the national patient registry. For each SLE patient, 10 age- and sex-matched controls without SLE and AMI before 1996 (n = 41 892) were identified. Data from patients and controls with AMI after 1996 were linked with the Swedish coronary angiography and angioplasty register; 549 (13%) and 3352 (8%) first AMIs occurred in patients with SLE and controls, respectively. The incidence of AMI was 9.6 (95% CI: 8.9-10.5) and 4.9 (95% CI: 4.8-5.1) events/1000 person-years in patients with SLE and controls, respectively. The proportion of MINOCA was 10.8% in patients with SLE and 13.8% in controls (*P* = .261), respectively. In conclusion, the incidence of AMI is increased in a European population of patients with SLE but there is no indication that the proportion of MINOCA is increased in these patients.

## Introduction

Systemic lupus erythematosus (SLE) is an autoimmune inflammatory disease with a wide range of clinical manifestations and complications.^1–3^ Cardiovascular disease (CVD) is substantially increased compared with the general population and is a well-recognized complication among patients with SLE.^4,5^ The increased CVD risk has been suggested to be multifactorial, with contributions from traditional CVD risk factors, SLE disease activity, and SLE-related medications.^6^ The prevalence of SLE in Sweden ranges from 46 to 85/100 000 inhabitants and occurs more commonly among women.^7^

During the first year after diagnosis, patients with SLE have an increased risk of CVD events as shown in a recent publication^5^ that assessed the risk of acute myocardial infarction (AMI), stroke, and CVD among 4863 patients with SLE in Canada showing an approximately 2-fold increased risk for all events. Immune complexes and presence of pro-inflammatory cytokines have been hypothesized to contribute to AMI by thrombotic occlusion of smaller arterial vessels.^8^ The risk for developing atherosclerotic lesions has also been reported to be increased in SLE.^2^ Atherosclerosis is suggested to be promoted by a complex interplay between autoantibodies and dysfunctional lipids that together with traditional CVD risk factors cause an inflammatory processes in the vascular wall.^9–11^ One retrospective cohort study evaluated the risk of AMI during the first 4 to 8 years after SLE diagnosis using Taiwan’s national health insurance research database. They identified 1207 patients newly diagnosed with SLE with 9656 age- and sex-matched non-SLE individuals as controls. Their conclusion was that patients with SLE had a 5-fold increased risk of AMI compared with controls.^12^

Considering that SLE is a lifelong inflammatory condition, previous studies are limited by a short follow-up time, and the incidence of AMI in patients with SLE has not yet been studied on a large scale in a European country. Furthermore, it has been suggested that myocardial ischemia among patients with SLE is connected to other mechanisms, such as microvascular dysfunction and antiphospholipid syndrome, rather than coronary artery atherothrombosis.^8,13^ The autoimmune inflammatory pathogenesis is also believed to mediate a variety of cardiac injuries from symptomatic pericarditis to myocarditis.^10,14^ It is today unknown whether patients with SLE, due to these factors, more frequently develop myocardial infarction (MI) with nonobstructive coronary arteries (MINOCA)^15^ compared with the general population.

The primary objective of the present study was to assess incident AMI cases, and the secondary objective was to estimate the proportion of MINOCA in patients with SLE.

## Materials and Methods

The present investigation was a register-based retrospective cohort study using the Swedish national inpatient register (NPR) and cause of death register (CDR), and the Swedish coronary angiography and angioplasty register (SCAAR). The cohort spanned from January 1, 1996, to December 31, 2015. We used the *International Classification of Diseases* (*ICD*) to define diseases and CVD risk factors (Table 1).

**Table 1.** *ICD* Codes for Diseases and Cardiovascular Risk Factors.

Diagnosis^a^	*ICD-10*	*ICD-9*	*ICD-8*
SLE	M32.0-32.9	710A	734.1
Acute myocardial infarction	I21.0-21.9	410A-X	410.0-410.99
Essential hypertension	I10.9	401A-X	400.0-401.99
Diabetes mellitus type 1 & 2	E10.0-10.9, E11.0-11.9	250	250.0-250.09
Renal disease	N00-N06, N082, N085, N162, N164, N168, N18.1-18.9, N19 Y60.2, Y84.1, Z99.2	580-586	580.00-584.99
Hyperlipidemia	E78.0, E78.1, E78.2, E78.4, E78.5	272, 272A, 272B, 272C, 272E, 272W	279.0-279.01
Obesity	E66.0-66.9	278, 278A, 278B	277.99
COPD	J44	496	491
Liver cirrhosis	K70.3, K74.6	571C, 571F	571
Mental disorders	F20, F31, F33, F41	295, 296, 300A, 311	295, 296, 300

Abbreviations: COPD, chronic obstructive pulmonary disease; *ICD, International Classification of Diseases*; SLE, systemic lupus erythematosus.

^a^ All diagnoses except for acute myocardial infarction (AMI) where defined if they occurred as main or secondary diagnosis in the national patient register (NPR). AMI was defined as main diagnosis in NPR or cause of death in the cause of death register. Mental disease was a combination of bipolar disease, including depression, anxiety syndromes, and schizophrenia.

### Patients With SLE and Controls

The initial cohort consisted of 4198 individuals with SLE without an AMI registered in the NPR before 1996. The patients were defined with the following criteria: They had been diagnosed with SLE according to *ICD-8* 734.1 or *ICD-9* 710A before 1996. They had not been diagnosed with an AMI according to *ICD-9*: 410 or *ICD-8*: 410, 411, 426, and 429 before 1996 and were not found in the CDR at that time. For every patient with SLE, 10 controls were included from the NPR, matched for age and gender. The comparison cohort was selected according to following criteria: Before 1996, they had not been diagnosed with AMI or SLE according to *ICD-9*: 410, 710A or *ICD-8*: 410, 411, 426, 429, and 734.1 and could not be found in the CDR at that time. A total of 90 individuals were excluded from the study. Two patients with SLE and 16 controls did not match the inclusion criteria and 72 controls were diagnosed with SLE during the follow-up period. The analyzed cohorts consisted of 4192 patients with SLE and 41 892 non-SLE individuals (Supplementary figure). Cardiovascular disease risk factors included essential hypertension, diabetes mellitus type 1 and 2, renal disease, hyperlipidemia, obesity, chronic obstructive pulmonary disease (COPD), liver cirrhosis, and mental disorders. Of the analyzed cohorts, 1218 individuals were registered in SCAAR. These patients had undergone coronary angiography 1 day before and up to 90 days after AMI diagnosis. Due to inconclusive result 69 events were excluded. In total, 1149 patients (29% of all patients with AMI) were included for analysis of prevalence of MINOCA (Supplementary figure). In SCAAR, obstruction of coronary arteries is defined as any presence of stenosis ≥50% irrespective of location and vessel distribution, whereas MINOCA is defined as a normal result or stenosis <50%. Inconclusive results were defined as inconclusive investigation or absence of results of a completed coronary angiography. The study was approved by the regional ethics committee (number 2016/1725-32) in Stockholm, Sweden.

### Statistical Methods

Descriptive data are shown in numbers (percentage) or mean ± standard deviation. Differences in proportions and means between the exposure groups (SLE vs non-SLE) were tested by χ^2^ tests or independent *t* test, respectively. To study survival time between the groups, we used the Kaplan-Meier method and corresponding log-rank test. In addition, to study the association between SLE and AMI after adjustments for known risk factors for CVD we used Cox regression. Time at risk was defined as time from baseline (1996) to first diagnosis of AMI (event), death of other cause than AMI (censoring), or end of follow-up (December 31, 2015) (censoring), which ever occurred first. Adjustments were made for hypertension, diabetes mellitus, renal disease, hyperlipidemia, obesity, COPD, liver cirrhosis, and mental disorders. We defined the risk factors at 2 different time points to study the influence on the associations between SLE and AMI depending on when a diagnosis for the risk factors first occurred (before 1996 or 1996 and later, ie, during the time at risk). All survival analyses were adjusted for sex and gender by the matched study design. We present the hazard ratio with corresponding 95% CI. The proportional hazard assumption was verified by studying the log-minus-log plot. All tests were 2 sided. The results were considered significant at *P* < .05. Analyses were carried out using SPSS versions 26 (IBM®).

## Results

### Incidence of AMI in SLE

The mean age of the study populations at baseline was 55 years; 83% were women. Compared with the comparison cohort, CVD risk factors, such as hypertension, diabetes mellitus, renal disease, COPD, liver cirrhosis, and mental disorders were more common in patients with SLE at baseline (Table 2). Among the 4192 patients with SLE, 549 (13.1%) experienced a first AMI during the 20-year follow-up period compared with 3352 (8.0%) in the comparison cohort. Patients with SLE experienced their AMI at an average age of 69 ± 13 years compared with 76 ± 11 years in the comparison cohort (*P* < .01).

**Table 2.** Patient Characteristics for Patients With SLE and Non-SLE Controls at Baseline.^a^

	SLE (n = 4 192)	Non-SLE (n = 41 892)	*P*
Age, years	55 ± 18	55 ± 18	NA
Female gender	3 487 (83.2)	34 848 (83.2)	NA
Hypertension	412 (9.8)	1642 (3.9)	**<.001**
Diabetes mellitus	219 (5.2)	1222 (2.9)	**<.001**
Renal disease	55 (1.3)	62 (0.1)	**<.001**
Hyperlipidemia	14 (0.3)	82 (0.2)	.061
Obesity	23 (0.5)	269 (0.6)	.464
COPD	66 (1.6)	167 (0.4)	**<.001**
Liver cirrhosis	31 (0.7)	80 (0.2)	**<.001**
Mental disorders	277 (6.6)	1885 (4.5)	**<.001**

Abbreviations: COPD, chronic obstructive pulmonary disease; NA, not applicable; SLE, systemic lupus erythematosus.

^a^ Values are n (%) or mean ± standard deviation. Bold *P* values denote significant differences. Diagnoses are based on *ICD* codes occurring before 1996 as defined in Table 1. Mental disease was a combination of bipolar disease, including depression, anxiety syndromes, and schizophrenia.

**Figure 1.**
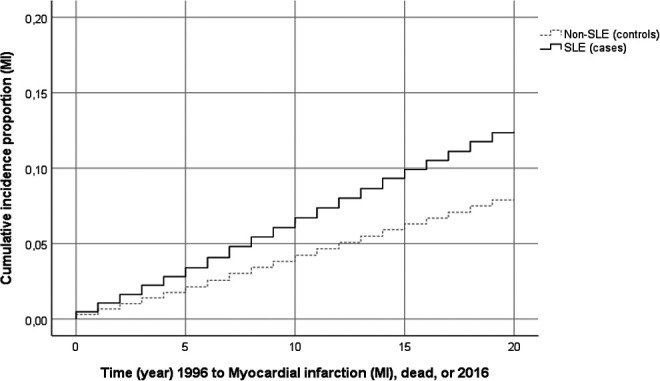
Survival analysis of the incidence of myocardial infarction (MI) in patients with systemic lupus erythematosus (SLE; n = 4192) and controls (n = 41 892) 1996 to 2016.

Systemic lupus erythematosus was associated with an increased incidence of AMI. Among the 4192 patients with SLE, the incidence of AMI was 9.6 (95% CI: 8.9-10.5) events/1000 person-years. In the comparison cohort, the incidence of AMI was 4.9 (95% CI: 4.8-5.1) events/1000 person-years. Survival analysis by Cox regression showed that the cumulative incidence of AMI was increased in SLE compared with controls (Figure 1). Of the CVD risk factors, a diagnosis of either hypertension, diabetes mellitus, COPD, or mental disorders before AMI may also have contributed to the increased AMI risk (Table 3).

**Table 3.** Unadjusted and Adjusted Hazard Ratios (HR) for the Risk of AMI in Patients With SLE (n = 4 192) Relative to the Comparison Cohort (n = 41 892).^a^

	Unadjusted	Adjusted for risk factors 1996	Adjusted for risk factors at AMI
SLE	1.9 (1.8-2.1)	1.7 (1.6-1.9)	1.6 (1.4-1.7)
Hypertension	3.9 (3.5-4.3)	2.9 (2.6-3.3)	11.7 (10.5-13.0)
Diabetes mellitus	4.4 (4.0-5.0)	3.2 (2.8-3.6)	2.8 (2.4-3.1)
Renal disease	1.3 (0.7-2.3)	0.9 (0.5-1.6)	1.1 (0.9-1.3)
Hyperlipidemia	2.9 (1.9-4.6)	1.2 (0.7-1.8)	0.9 (0.7-1.01)
Obesity	1.6 (1.2-2.2)	1.0 (0.7-1.4)	1.2 (0.9-1.6)
COPD	2.5 (1.7-3.6)	1.7 (1.2-2.5)	1.6 (1.3-1.9)
Liver cirrhosis	1.6 (0.9-2.8)	1.1 (0.6-2.0)	0.8 (0.5-1.2)
Mental disorders	1.3 (1.2-1.6)	1.3 (1.1-1.4)	2.1 (1.8-2.5)

Abbreviations: AMI, acute myocardial infarction; COPD, chronic obstructive pulmonary disease; SLE, systemic lupus erythematosus.

^a^ Values are HR (95% CIs). All analyses (unadjusted and adjusted) are adjusted for age and gender through the matched study design.

### Proportion of MINOCA in SLE

The mean age of first observed AMI with a coronary angiography was lower in SLE patients (63 years) than in the control group (70 years). The distribution of men and women and risk factors for CVD were similar in both groups with the exception for renal disease that was more common among patients with SLE (Table 4). For patients with SLE, 20 (10.8%) of 186 had MINOCA, whereas 133 (13.8%) of 963 in the comparison cohort had MINOCA (*P* = .261).

**Table 4.** Patient Characteristics for Patients With SLE and Non-SLE Controls at the Time of AMI Investigated by Coronary Angiography.^a^

	SLE (n = 186)	Non-SLE (n = 963)	*P*
Age, years^b^	63 ± 11	70 ± 10	**<.001**
Female gender	142 (76.3)	692 (71.9)	.209
Hypertension	63 (33.9)	279 (29.0)	.189
Diabetes mellitus	23 (12.4)	141 (14.6)	.427
Renal disease	29 (15.6)	9 (0.9)	**<.001**
Hyperlipidemia	17 (9.1)	82 (8.5)	.887
Obesity	3 (1.6)	21 (2.2)	.784
COPD	11 (5.9)	50 (5.2)	.720
Liver cirrhosis	3 (1.6)	4 (0.4)	.089
Mental disorders	24 (12.9)	80 (8.3)	.051

Abbreviations: AMI, acute myocardial infarction; COPD, chronic obstructive pulmonary disease; MINOCA, myocardial infarction with nonobstructive coronary arteries; SLE, systemic lupus erythematosus.

^a^ Values are n (%) or mean ± standard deviation. Bold *P* values denote significant differences. Diagnoses are based on *ICD* codes.

^b^ Age at the first AMI with a coronary angiography investigation 1 day before or 90 days after diagnosis.

There were no differences in age, sex distribution, and CVD risk factors between MINOCA patients with the exception for renal disease that was more common among patients with SLE. Among AMI patients with obstructive coronary arteries, mean age was lower, renal disease, liver cirrhosis, and mental disorder more common in patients with SLE (Table 5).

**Table 5.** Patient Characteristics for Patients With SLE and Non-SLE Controls at the Time of AMI Investigated By Coronary Angiography Separated By Coronary Angiography Results.^a^

	MINOCA			Obstructive CAD		
	SLE (n = 20)	Non-SLE (n = 133)	*P*	SLE (n = 166)	Non-SLE (n = 830)	*P*
Age, years^b^	63±12	66±11	0.158	63±11	71±10	**<.001**
Female gender	19 (95.0)	117 (88.0)	0.351	123 (74.1)	575 (69.3)	.216
Hypertension	4 (20.0)	35 (26.3)	0.600	59 (35.5)	244 (29.4)	.139
Diabetes mellitus	2 (10.0)	12 (9.0)	0.999	21 (12.7)	129 (15.5)	.405
Renal disease	3 (15.0)	2 (1.5)	**0.016**	26 (15.7)	7 (0.8)	**<.001**
Hyperlipidemia	0 (0)	6 (4.5)	0.602	17 (10.2)	76 (9.2)	.771
Obesity	0 (0)	5 (3.8)	0.619	3 (1.8)	16 (1.9)	.999
COPD	2 (10.0)	7 (5.3)	0.333	9 (5.4)	43 (5.2)	.850
Liver cirrhosis	0 (0)	2 (1.5)	0.999	3 (1.8)	2 (0.2)	**.035**
Mental disorders	2 (10.0)	15 (11.3)	0.999	22 (13.3)	65 (7.8)	**.034**

Abbreviations: AMI, acute myocardial infarction; CAD, coronary artery disease; COPD, chronic obstructive pulmonary disease; MINOCA, myocardial infarction with nonobstructive coronary arteries; SLE, systemic lupus erythematosus.

^a^ Values are n (%) or mean ± standard deviation. Bold *P* values denote significant differences. Diagnoses are based on *ICD* codes.

^b^ Age at the first AMI with a coronary angiography investigation 1 day before or 90 days after diagnosis.

## Discussion

The results of the present register-based study showed that the incidence of AMI was doubled in patients with SLE when compared with an age- and sex-matched cohort without SLE. There were no differences regarding the proportion of patients with MINOCA between patients with SLE and controls.

### Incidence of AMI in SLE

There are 2 register-based studies showing an increased incidence of AMI in patients with SLE when compared with cohorts without SLE.^5,12^ The incidence of AMI in patients with SLE was lower in both studies when compared with our results but the magnitude of the difference between patients and controls was similar to the study from Canada.^5^ The causes for the higher incidence in our study could be an older SLE cohort, a longer follow-up and that we only included patients treated at in-hospital care. Another difference that might have affected the results is that both the Canadian^5^ and the Taiwanese^12^ studies followed incident cases whereas we studied prevalent cases with a confirmed SLE diagnosis any time before 1996. This possibly underestimated the risk of AMI in our study since the risk is highest after the diagnosis of SLE. Furthermore, the number of patients with SLE with AMI in our study was considerably higher than the Canadian (<5 times)^5^ and Taiwanese (>15 times)^12^ studies, respectively. Taken together, we could confirm an increased incidence of AMI in patients with SLE. We also extend the results from previous register-based studies by showing this association in a European population. The present SLE cohort also had a considerably longer follow-up (20 years for all patients) compared with the Canadian (10-15 years)^5^ and Taiwanese (4-8 years)^12^ populations, respectively, thus also showing an increased long-term risk for AMI. The reason for the increased incidence of AMI is possibly a combination of SLE-related factors, such as immune-complexes, immune-modulatory drugs and inflammation, and traditional CVD risk factors.^8,9^ This is supported by the contribution of hypertension, diabetes mellitus, and COPD to the increased risk.

### Myocardial Infarction With Nonobstructive Coronary Arteries in SLE

MINOCA has been increasingly recognized due to the common use of high-sensitive troponins and coronary angiography.^15^ According to American and European recommendations, MINOCA is considered a working diagnosis requiring further investigations with cardiac magnetic resonance imaging among other investigations. Furthermore, both recommendations emphasize the need to exclude causes of spontaneous thromboembolism such as the antiphospholipid syndrome.^16,17^ There are no systematic studies of MINOCA in SLE but a case report of 2 patients suggested an association between SLE and MINOCA.^18^ There is only 1 systematic study of the antiphospholipid syndrome comprising 25 MINOCA patients showing no patients with lupus anticoagulant or raised anticardiolipin antibodies compared with patients with MINOCA.^19^ The results of the present study speak against MINOCA as a major cause of AMI in SLE and suggest that the majority of cases are attributed to atherothrombosis. Since we studied prevalent SLE cases, it is not possible to extrapolate the findings to incident SLE cases shown to have an increased risk for AMI the year after diagnosis.^5^ The results thus argue against misdiagnosis of AMI by antiphospholipid syndrome, microvascular dysfunction, and myocarditis.^10,13,14^ However, to firmly make a MI diagnosis in SLE, systematic studies using cardiac magnetic resonance imaging are warranted.

### Strengths and Limitations

The strengths of the present study are the large size and long follow-up of the patients. It also included all Swedish patients with an SLE diagnosis before 1996 and for the first time showed coronary angiography findings in this patient group.

The main limitation is the retrospective register-based design with a small number of possible confounders and low internal validity. There is lack of information about socioeconomic and lifestyle confounders, for example, smoking, and the validity of the SLE and AMI diagnoses. Chronic obstructive pulmonary disease was used as a proxy for smoking but cannot fully compensate for the lack of smoking data. The validity of the SLE diagnoses can be questioned since the SLE diagnoses were made before 1996 and have not been validated. However, misdiagnoses are unlikely since we included SLE diagnoses made in an in-hospital population with a presumably severe disease, whereas the validity of the diagnosis AMI has been validated.^20^ Another limitation is that we included prevalent cases at the end of 1995 and could thus not study the immediate risk of AMI associated with the acute onset of SLE.

## Conclusions

The incidence of AMI is increased in a European population of patients with SLE but there is no indication that the proportion of MINOCA is increased in SLE. The practical implication of the present study results is that it is important to investigate patients with SLE with AMI with coronary angiography. However, to firmly make a MI diagnosis in SLE, systematic use of cardiac magnetic resonance imaging is warranted.

## Supplemental Material

Supplemental Material, sj-pdf-1-ang-10.1177_0003319720985337 - Myocardial Infarction in Systemic Lupus Erythematosus: Incidence and Coronary Angiography Findings Click here for additional data file. Supplemental Material, sj-pdf-1-ang-10.1177_0003319720985337 for Myocardial Infarction in Systemic Lupus Erythematosus: Incidence and Coronary Angiography Findings by Per Tornvall, Alexandra Göransson, Julia Ekman and Hans Järnbert-Pettersson in Angiology
