# Supplementary material for: Myocardial Infarction in Systemic Lupus Erythematosus: Incidence and Coronary Angiography Findings
Source: Angiology. 2021 Jan 8;72(5):459–64. doi: 10.1177/0003319720985337 (PMC8044619; doi:10.1177/0003319720985337)

Supplementary Figure. Consort diagram of included patients and controls
AMI=acute myocardial infarction; ICD = international statistical classification of diseases; MINOCA = myocardial infarction with non-obstructive coronary arteries; NPR= national patient register; PCI = percutaneous coronary intervention; SCAAR = Swedish coronary angiography and angioplasty register; SLE = systemic lupus erythematosus.

Supplementary figure

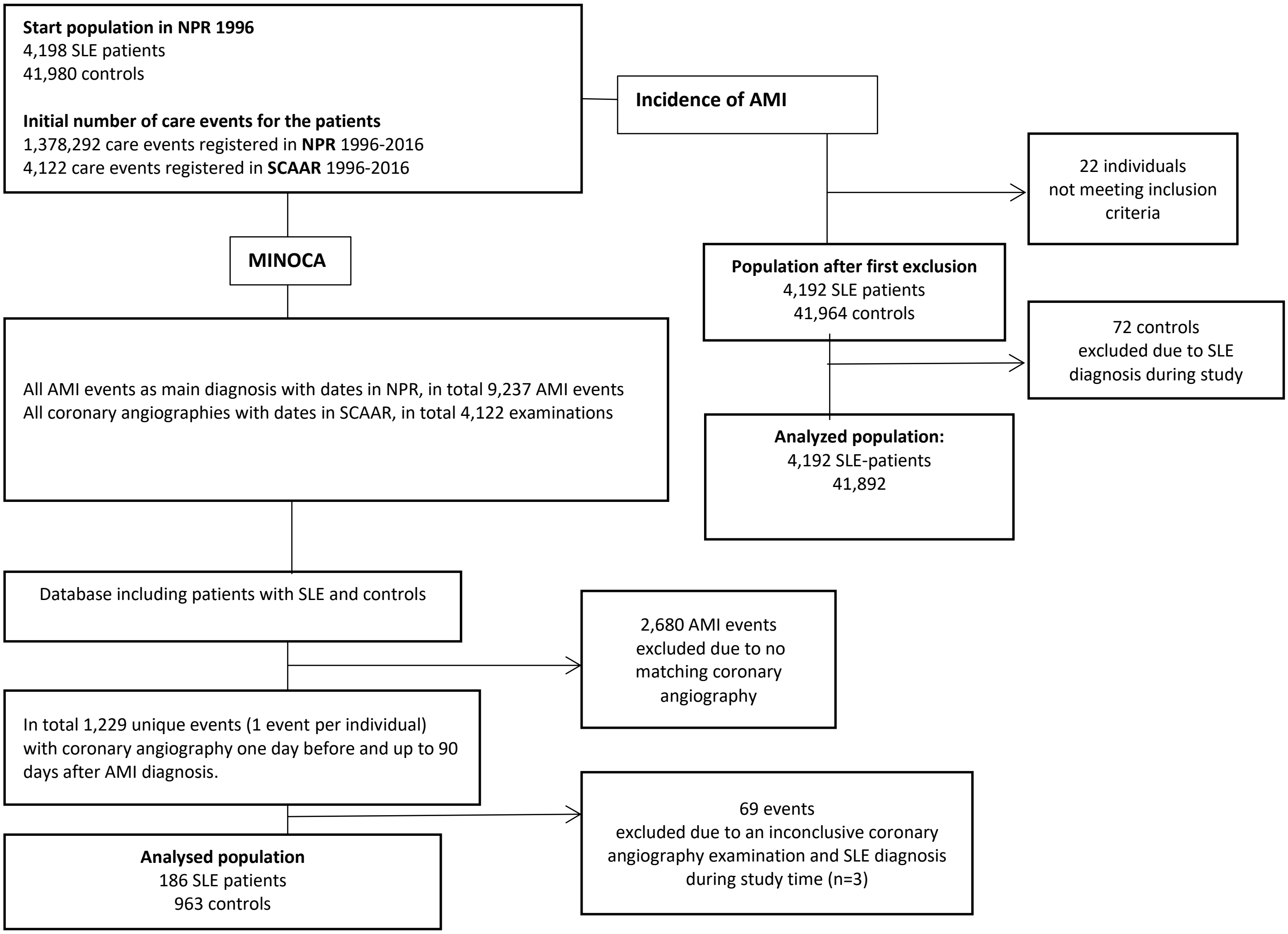

Supplement: Supplemental Material, sj-pdf-1-ang-10.1177_0003319720985337 - Myocardial Infarction in Systemic Lupus Erythematosus: Incidence and Coronary Angiography Findings [file sj-pdf-1-ang-10.1177_0003319720985337.pdf]
